# Comparative Analysis of Doxorubicin-Induced Cardiotoxicity in Tumor-Bearing and Non-Tumor-Bearing Mouse Models: A Transcriptomic Methodological Study

**DOI:** 10.3390/ijms27125263

**Published:** 2026-06-10

**Authors:** Aonan Yu, Rong Yang, Yaojiang Wang, Peng Yang, Xinyu Liu, Jingjing Guo, Haoyu Xia, Liliang Yang, Mengxiao Zhang

**Affiliations:** 1 Department of Pharmacy, Bengbu Medical University, Bengbu 233030, China; y15055860069@163.com (A.Y.); sawazure@163.com (R.Y.); 17856838558@163.com (Y.W.); ypeng5454@163.com (P.Y.); 18355707020@163.com (X.L.); guojingjing0811@126.com (J.G.); 19335284487@163.com (H.X.); 2 Anhui Engineering Technology Research Center of Biochemical Pharmaceutical, Bengbu Medical University, Bengbu 233030, China

**Keywords:** doxorubicin, tumor, cardiotoxicity, transcriptomic analysis, animal model

## Abstract

Doxorubicin (DOX) is a widely used anthracycline chemotherapeutic agent whose clinical application is limited by cardiotoxicity. In clinical settings, chemotherapy is given to tumor-bearing patients, whereas most preclinical studies of DOX-related cardiotoxicity use non-tumor-bearing animal models, potentially missing context-dependent differences. To address this, we compared DOX-induced cardiotoxicity between non-tumor-bearing and tumor-bearing mouse models. Cardiac function was assessed by echocardiography, and serum biomarkers, histopathological changes, and cardiac transcriptomic profiles were analyzed. Tumor burden exacerbated DOX-induced increases in BNP and CK-MB levels and myocardial structural damage, whereas systolic function was significantly reduced in non-tumor-bearing mice but did not further decline in tumor-bearing mice. Transcriptomic analysis revealed that DOX treatment induced 2528 and 398 differentially expressed genes (DEGs) in non-tumor-bearing and tumor-bearing mice, respectively, compared with their respective controls. A total of 206 shared DEGs were identified, most of which showed consistent directions of change under both conditions, while 16 genes exhibited opposite expression patterns. Common DEGs were mainly enriched in immune-inflammatory responses, cell adhesion, and extracellular matrix (ECM)–receptor interaction pathways. In non-tumor-bearing conditions, DOX-specific mechanisms were mainly associated with ECM remodeling, oxidative stress, metabolic dysregulation, and p53-mediated apoptosis. In contrast, tumor-bearing conditions showed predominant enrichment of immune-related pathways, including JAK-STAT, Toll-like receptor, NOD-like receptor, and chemokine signaling. These findings suggest that tumor burden may modulate the molecular mechanisms of DOX-induced cardiotoxicity, revealing context-dependent differences and offering insights for future cardioprotective strategies.

## 1. Introduction

Doxorubicin (DOX) is a chemotherapeutic agent extensively used in the treatment of both solid tumors and hematological malignancies [1]. However, its clinical utility is significantly limited by dose-dependent and potentially life-threatening cardiotoxicity. DOX-induced cardiotoxicity may result in cardiac dysfunction and cardiomyopathy, ultimately progressing to heart failure and mortality [2]. The canonical molecular mechanisms underlying this toxicity have been predominantly elucidated in studies employing healthy animal models, which have systematically characterized the direct deleterious effects of DOX on cardiomyocytes, including the inhibition of topoisomerase IIβ and the induction of mitochondrial oxidative stress [3,4]. The reliance on healthy animal models is largely attributable to their relatively stable and well-controlled genetic background, metabolic profile, and immune milieu, which reduce the confounding influences introduced by tumor burden and thereby facilitate the dissection of the fundamental mechanisms of drug-induced toxicity [5]. However, such models do not reflect the clinical reality, where patients receiving DOX-based chemotherapy are also under tumor burden.

Tumors per se can modulate cardiac structure and function through multiple mechanisms. Tumor growth adjacent to or within cardiac tissue may exert mechanical compression, leading to myocardial deformation and cardiac dysfunction [6]. In addition, tumor-derived bioactive mediators, including inflammatory cytokines, chemokines, and reactive oxygen species (ROS), can induce systemic inflammation and metabolic dysregulation, thereby promoting myocardial changing, such as atrophy and fibrosis, and impairing cardiac function. Tumor-driven metabolic reprogramming may also interact with DOX-induced mitochondrial dysfunction and oxidative stress, collectively affecting cardiomyocyte energy metabolism and cell death pathways, including ferroptosis [7]. Tumor-derived extracellular vesicles, particularly exosomes, can further mediate intercellular communication by delivering bioactive molecules (e.g., non-coding RNAs and proteins) to cardiomyocytes, thereby regulating gene expression and cellular stress responses [8].

Therefore, mechanistic insights into DOX-induced cardiotoxicity derived from healthy models may not fully recapitulate the complex network of drug toxicity under tumor-bearing conditions. To address this knowledge gap, the present study employed a comparative analytical strategy to systematically characterize the cardiac transcriptomic responses to DOX in both healthy and tumor-bearing mouse models. By directly comparing gene expression profiles and pathway enrichment signatures between these two contexts, this work aims to establish a more precise molecular framework for understanding DOX-induced cardiotoxicity in cancer patients and to provide new perspectives for developing context-specific cardioprotective strategies.

## 2. Results

### 2.1. Effects of Doxorubicin on Cardiac Function and Serum Biomarkers in Non-Tumor-Bearing and Tumor-Bearing States

**Figure 1:**
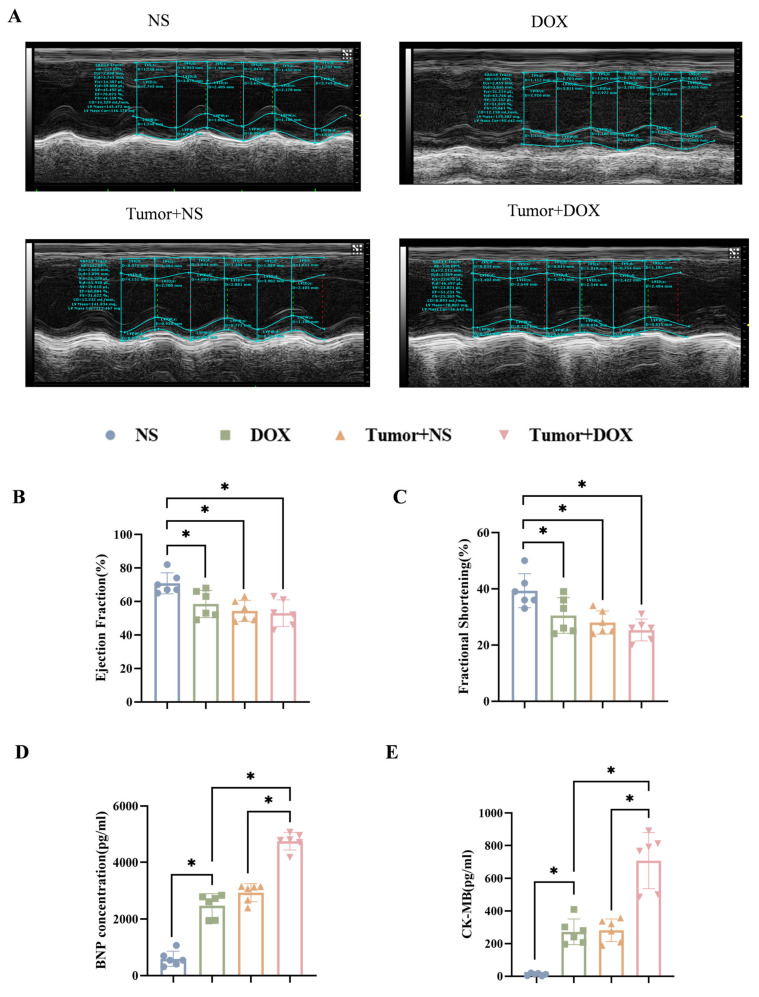
Effects of doxorubicin on cardiac injury under non-tumor-bearing and tumor-bearing conditions. For all graphs, *n* = 6 mice per group. (**A**) Representative M-mode echocardiographic images. Green dash: End-systolic left ventricular internal dimension. Red dash: End-diastolic left ventricular internal dimension. (**B**) Ejection fraction (EF). (**C**) Fractional shortening (FS). (**D**) Serum BNP levels in each group. (**E**) Serum CK-MB levels in each group. * *p* < 0.05.

To evaluate the cardiotoxic effects of DOX under both tumor-bearing and non-tumor-bearing conditions, cardiac function was assessed using echocardiography. Compared with the normal saline (NS)-treated control, DOX impaired cardiac systolic function in both non-tumor-bearing and tumor-bearing mice, as evidenced by comparable reductions in ejection fraction (EF) and fractional shortening (FS) under both conditions (Figure 1A–C). Cardiac injury was further evaluated by measuring serum biomarkers of myocardial damage. Serum B-type natriuretic peptide (BNP) and creatine kinase-MB (CK-MB) levels were significantly elevated in tumor-bearing mice even at baseline (NS-treated) compared with non-tumor-bearing controls. Following DOX treatment, both markers increased further, and their levels in tumor-bearing DOX-treated mice were significantly higher than those in non-tumor-bearing DOX-treated mice (Figure 1D,E), suggesting greater cardiac stress and myocardial injury under tumor-bearing conditions.

### 2.2. Effects of Doxorubicin on Cardiac Structural Remodeling in Non-Tumor-Bearing and Tumor-Bearing States

**Figure 2:**
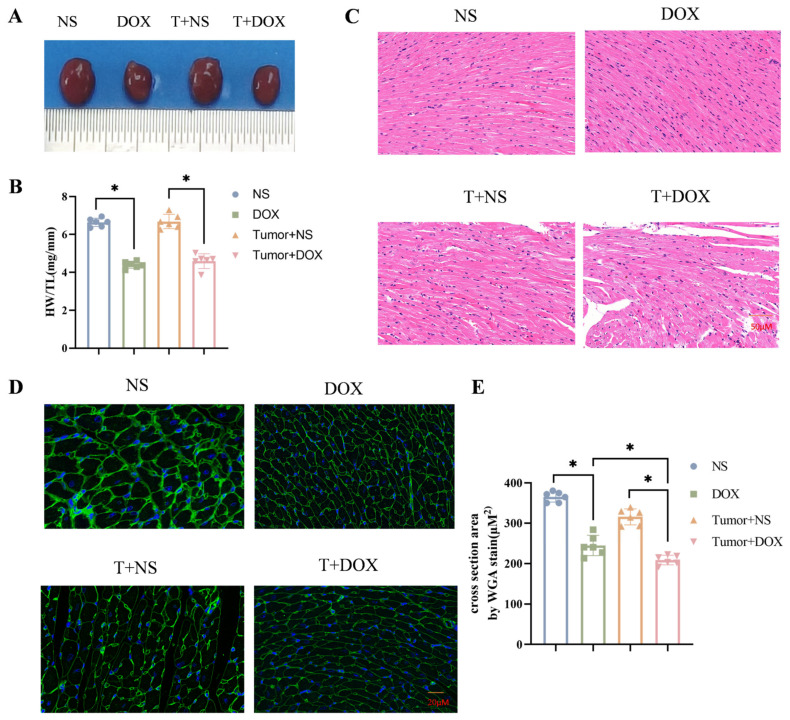
Effects of doxorubicin on cardiac structure under tumor-bearing conditions. For all graphs, *n* = 6 mice per group. (**A**,**B**) Representative images showing heart size and the heart weight-to-tibia length ratio in each group. (**C**) Representative hematoxylin and eosin (HE) staining of heart tissues. (**D**) Representative wheat germ agglutinin (WGA) staining of heart sections. (**E**) Quantification of cardiomyocyte cross-sectional area (CSA) using ImageJ software (version 1.54f). Data are presented as mean ± SD. * *p* < 0.05.

Based on the observed phenotypic differences, histological changes in myocardial tissue were further examined by hematoxylin and eosin (H&E) staining across different groups. Variations in overall tissue integrity and cellular distribution were observed among the treatment groups. Morphological analysis of the heart revealed differences in heart size (Figure 2A). Quantitative assessment of cardiac mass, expressed as the heart weight-to-tibia length ratio, showed a decrease in DOX-treated non-tumor-bearing mice compared with controls; a similar reduction was observed in DOX-treated tumor-bearing mice (Figure 2B), indicating that DOX induced structural alterations under both conditions. Histopathological examination further demonstrated that, compared with the non-tumor-bearing condition, myocardial tissue from DOX-treated tumor-bearing mice exhibited increased inflammatory cell infiltration and localized myocardial disarray, suggesting more pronounced structural damage in the presence of tumor burden (Figure 2C). WGA staining revealed significant differences in cardiomyocyte cross-sectional area (CSA) among groups (Figure 2D,E). Compared with the non-tumor-bearing group, the tumor-bearing group showed reduced CSA. DOX treatment further decreased CSA in both groups relative to their respective controls. Notably, CSA in the tumor-bearing DOX-treated group was significantly smaller than that in the non-tumor-bearing DOX-treated group, indicating that tumor burden may exacerbate DOX-induced structural remodeling of cardiomyocytes.

### 2.3. Differential Gene Expression Induced by Doxorubicin in Tumor-Bearing and Non-Tumor-Bearing States

**Figure 3:**
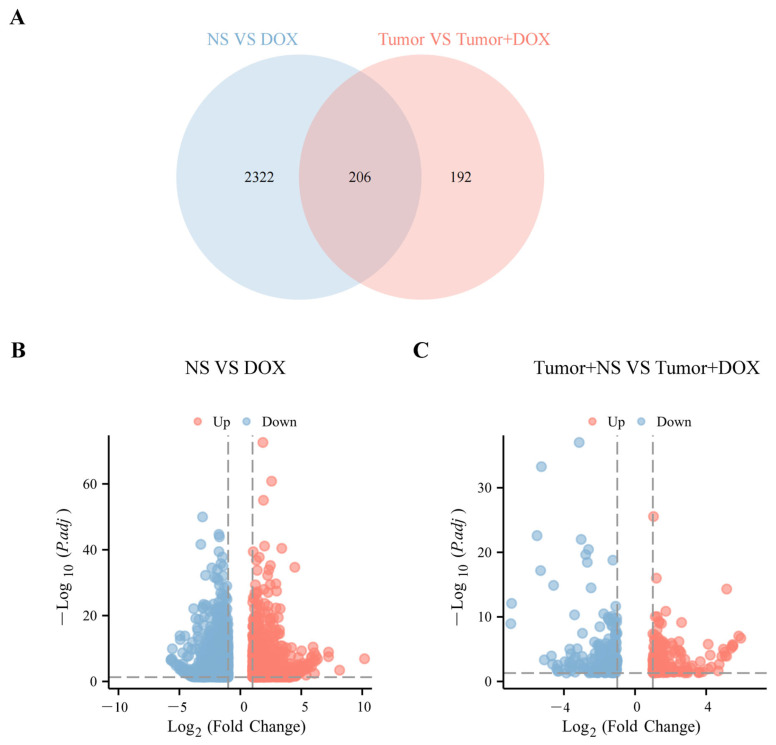
Effects of doxorubicin on differential gene expression under tumor-bearing and non-tumor-bearing conditions. (**A**) Venn diagram showing the overlap of differentially expressed genes (DEGs) among the indicated groups. Numbers represent the number of DEGs in each group. (**B**) Upregulated and downregulated DEGs in the NS vs. DOX comparison. (**C**) Upregulated and downregulated DEGs in the tumor-bearing NS vs. tumor-bearing DOX comparison.

To systematically compare the cardiac transcriptional alterations induced by doxorubicin under tumor-bearing and non-tumor-bearing conditions, transcriptome sequencing and differential expression analysis were performed on heart tissues from both models. The results showed that doxorubicin treatment induced widespread changes in cardiac gene expression, indicating a substantial impact on transcriptional profiles. Venn diagram analysis identified 2528 differentially expressed genes (DEGs) in non-tumor-bearing mice and 398 DEGs in tumor-bearing mice following doxorubicin treatment (Figure 3A). The expression patterns of DEGs differed markedly between the two conditions. In non-tumor-bearing mice, doxorubicin treatment resulted in 1193 upregulated and 1335 downregulated genes (Figure 3B), whereas in tumor-bearing mice, 187 upregulated and 211 downregulated genes were identified (Figure 3C). Compared with the non-tumor-bearing condition, DEGs in tumor-bearing mice exhibited distinct expression patterns, suggesting an altered transcriptional response of cardiac tissue to doxorubicin under tumor-bearing conditions. These findings indicate that tumor burden may reshape doxorubicin-induced cardiac transcriptional regulation.

### 2.4. Analysis of Shared DEGs in the Two Model Conditions

**Figure 4:**
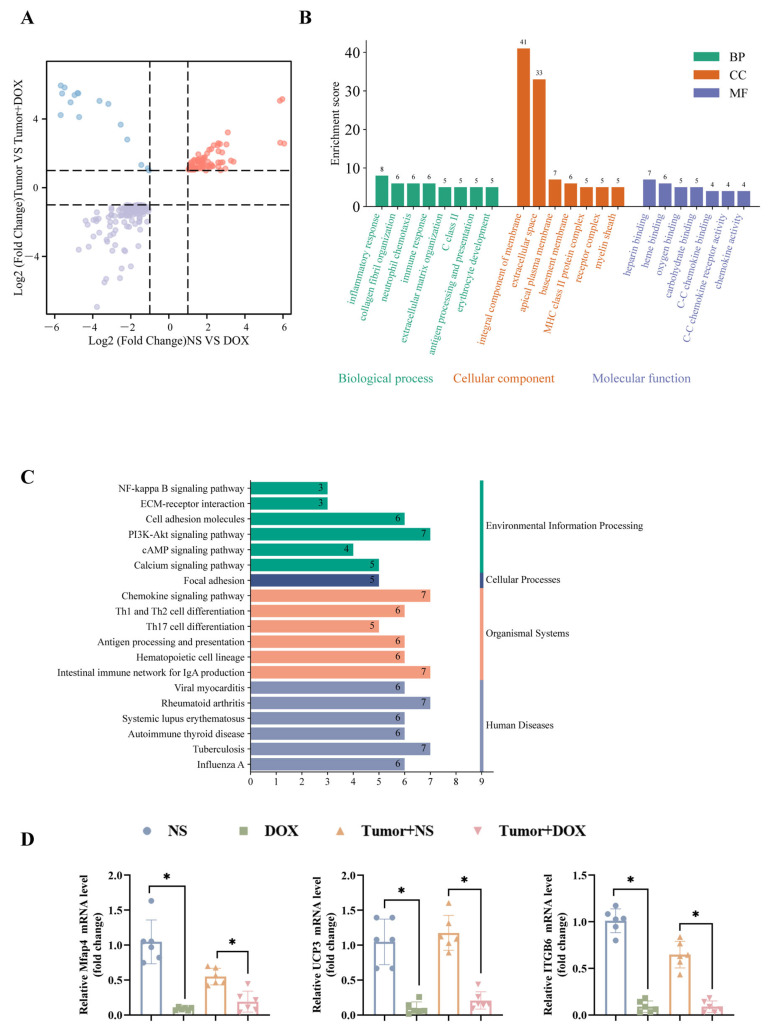
Transcriptomic analysis of shared differentially expressed genes under tumor-bearing and non-tumor-bearing conditions following doxorubicin treatment. (**A**) Nine-quadrant plot showing the expression trends of the 206 shared DEGs between non-tumor-bearing and tumor-bearing conditions. (**B**) Gene Ontology (GO) enrichment analysis of shared differentially expressed genes (DEGs). (**C**) Kyoto Encyclopedia of Genes and Genomes (KEGG) pathway enrichment analysis of shared DEGs. (**D**) qPCR validation of Mfap4, ITGB6, and UCP3 expression levels in heart tissues. * *p* < 0.05.

To investigate the common molecular mechanisms underlying doxorubicin-induced cardiotoxicity under non-tumor-bearing and tumor-bearing conditions, Gene Ontology (GO) and Kyoto Encyclopedia of Genes and Genomes (KEGG) enrichment analyses were performed on the shared DEGs. Expression pattern analysis of the 206 shared DEGs revealed that most genes displayed similar upregulation or downregulation trends under both conditions. However, 16 DEGs exhibited opposite expression patterns between the non-tumor-bearing and tumor-bearing groups (Figure 4A). GO enrichment analysis showed that the shared DEGs were primarily enriched in biological process (BP) terms, including positive regulation of cell–cell adhesion, immune response, inflammatory response, and cytokine-mediated signaling pathways. In terms of cellular component (CC), these genes were mainly associated with the extracellular space, integral component of membrane, and collagen-containing extracellular matrix. For molecular function (MF), enrichment was mainly observed in outward rectifier potassium channel activity, transmembrane signaling receptor binding, and receptor binding (Figure 4B). KEGG pathway analysis indicated that the shared DEGs were mainly enriched in immune- and adhesion-related pathways, including the NF-κB signaling pathway, chemokine signaling pathway, Th1 and Th2 cell differentiation, Th17 cell differentiation, antigen processing and presentation, cell adhesion molecules, ECM–receptor interaction, focal adhesion, and the PI3K-Akt signaling pathway (Figure 4C). From the shared DEGs, three representative genes (*Mfap4*, *ITGB6*, and *UCP3*) were selected for quantitative real-time PCR (qPCR) validation (Figure 4D). The qPCR results confirmed the transcriptomic findings. Collectively, these results indicate that shared DEGs are mainly involved in immune and inflammatory responses, cell adhesion, and extracellular matrix-related processes under both conditions.

### 2.5. Analysis of DEGs and Functional Enrichment Specific to Doxorubicin in the Non-Tumor-Bearing State

**Figure 5:**
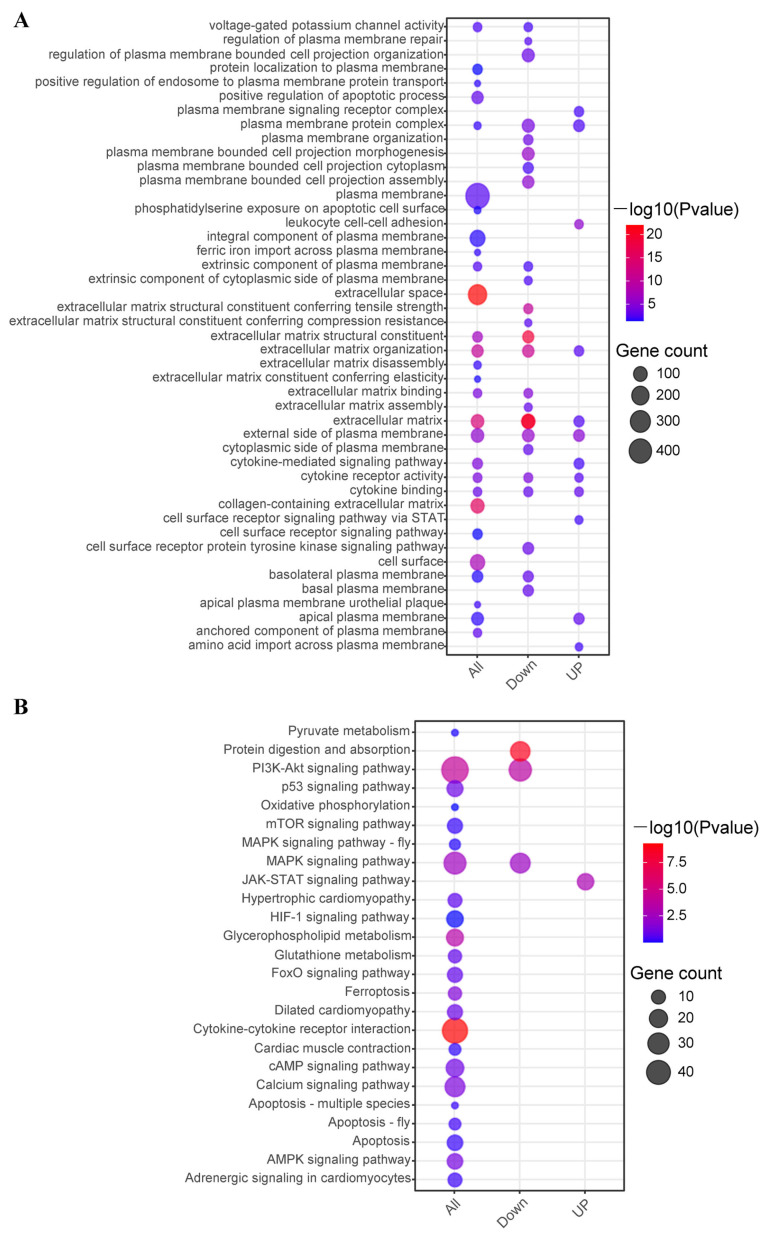
Transcriptomic analysis of doxorubicin under non-tumor-bearing conditions. (**A**) Gene Ontology (GO) enrichment analysis of differentially expressed genes (DEGs). (**B**) Kyoto Encyclopedia of Genes and Genomes (KEGG) pathway enrichment analysis of DEGs.

To systematically compare transcriptomic responses induced by DOX under tumor-bearing and non-tumor-bearing conditions, KEGG pathway and GO functional enrichment analyses were performed on DEGs identified in each group. The results revealed distinct molecular responses between the two conditions. We first focused on DEGs that were specifically altered only in the non-tumor-bearing state. GO enrichment analysis showed that these non-tumor-bearing-specific DEGs were mainly enriched in BP terms related to extracellular matrix organization, cytokine-mediated signaling pathways, and amino acid import across plasma membrane. In terms of CC, these genes were mainly localized to the external side of plasma membrane and plasma membrane structures. For MF, enrichment was observed in extracellular matrix structural constituent and extracellular matrix binding (Figure 5A). When these DEGs were further stratified by expression direction, distinct patterns emerged. Downregulated genes were mainly enriched in terms associated with cytokine-mediated signaling pathways and the external side of the plasma membrane. In contrast, upregulated genes were predominantly enriched in extracellular matrix organization, amino acid import across plasma membrane, plasma membrane structures, extracellular matrix structural constituent, and extracellular matrix binding (Figure 5A).

KEGG pathway analysis revealed that these non-tumor-bearing-specific DEGs were mainly enriched in metabolic pathways, including fatty acid metabolism, glutathione metabolism, and the pentose phosphate pathway, as well as signaling pathways such as PI3K–Akt, MAPK, JAK-STAT and AMPK signaling. Apoptosis and the p53 signaling pathway were also significantly enriched (Figure 5B). Stratified KEGG pathway analysis of upregulated and downregulated DEGs within this specific set revealed distinct enrichment patterns. Downregulated DEGs were mainly enriched in protein digestion and absorption, PI3K–Akt signaling pathway, and MAPK signaling pathway, whereas upregulated DEGs were primarily enriched in the JAK–STAT signaling pathway (Figure 5B). Taken together, these results suggest that DOX-induced cardiotoxicity under non-tumor-bearing conditions involves extracellular matrix remodeling, inflammatory responses, and metabolic and signaling pathway dysregulation.

### 2.6. Analysis of Differentially Expressed Genes and Functional Enrichment Specific to Doxorubicin in the Tumor-Bearing State

**Figure 6:**
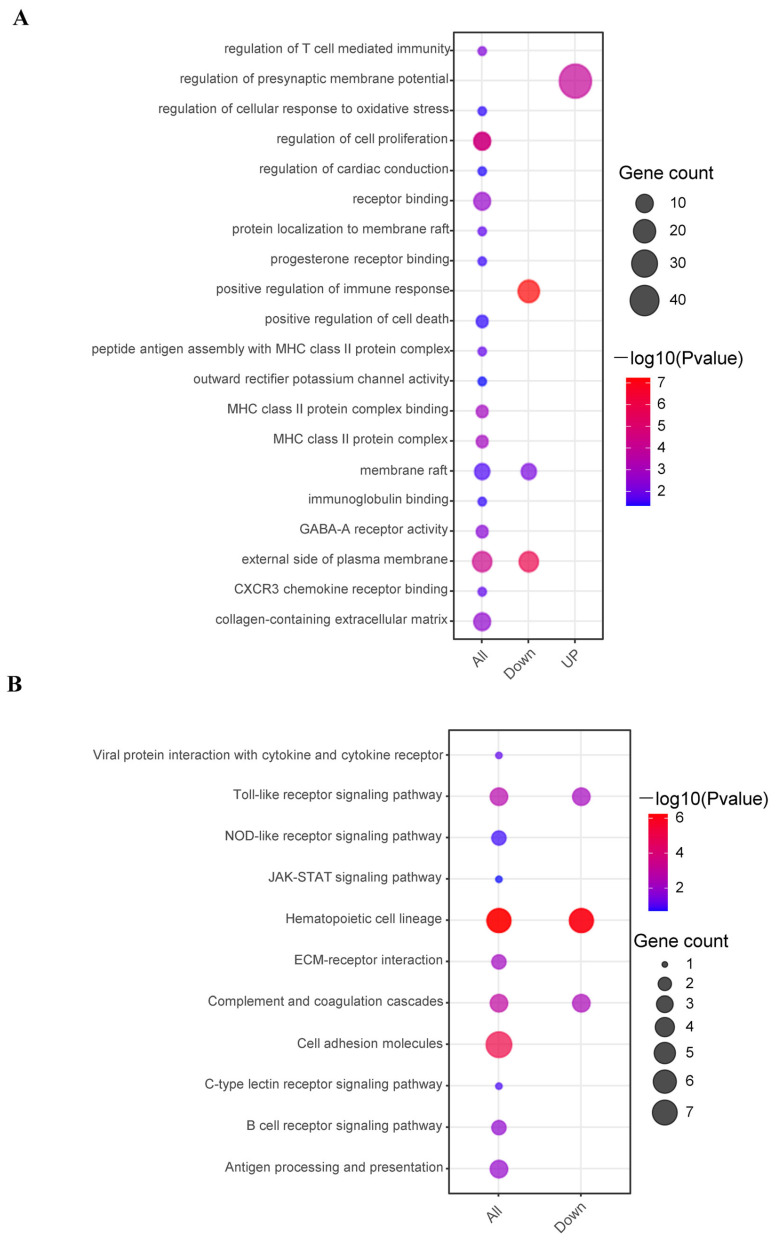
Transcriptomic analysis of doxorubicin under tumor-bearing conditions. (**A**) Gene Ontology (GO) enrichment analysis of differentially expressed genes (DEGs). (**B**) Kyoto Encyclopedia of Genes and Genomes (KEGG) pathway enrichment analysis of DEGs.

To investigate the specific mechanisms of doxorubicin under tumor-bearing conditions, GO and KEGG enrichment analyses were performed on tumor-bearing-specific DEGs. GO enrichment analysis of these DEGs revealed that upregulated genes were mainly enriched in regulation of presynaptic membrane potential, whereas downregulated genes were primarily associated with positive regulation of immune response. Other enriched biological processes included regulation of T cell-mediated immunity, oxidative stress response, cell proliferation, cardiac conduction, and cell death. In the cellular component category, DEGs were enriched in collagen-containing extracellular matrix, while downregulated genes were specifically enriched in the membrane raft and external side of plasma membrane. In the molecular function category, enriched terms mainly included receptor binding, outward rectifier potassium channel activity, and CXCR3 chemokine receptor binding (Figure 6A).

KEGG pathway enrichment analysis of these tumor-bearing specific DEGs showed that the enriched pathways were mainly associated with immune regulation and tumor–microenvironment interactions. Specifically, enriched pathways included viral protein interaction with cytokine and cytokine receptor, Toll-like receptor signaling pathway, NOD-like receptor signaling pathway, JAK-STAT signaling pathway, hematopoietic cell lineage, ECM–receptor interaction, complement and coagulation cascades, cell adhesion molecules, C-type lectin receptor signaling pathway, B cell receptor signaling pathway, and antigen processing and presentation. When these DEGs were stratified by expression direction, the Toll-like receptor signaling pathway, hematopoietic cell lineage, and complement and coagulation cascades were specifically enriched among downregulated genes, whereas no KEGG pathways were significantly enriched in upregulated genes. (Figure 6B). These findings suggest that tumor-bearing-specific DEGs are mainly involved in immune-inflammatory responses, cytokine signaling, and extracellular matrix organization-molecular changes that are distinct from those observed in the non-tumor-bearing state.

## 3. Discussion

In the present study, we compared DOX-induced cardiotoxicity between tumor-bearing and non-tumor-bearing mouse models. Our results showed that although DOX similarly impairs cardiac systolic function in both models, tumor-bearing mice exhibit more severe myocardial structural damage and higher serum biomarker levels. Transcriptomic analysis revealed that shared gene expression changes between the two models are mainly related to immune-inflammatory responses and extracellular matrix remodeling. Notably, non-tumor-bearing mice showed enrichment in oxidative stress, metabolic dysregulation, and p53-mediated apoptosis, whereas tumor-bearing mice were characterized by enrichment of immune-related pathways such as JAK–STAT, Toll-like receptor, and chemokine signaling. These findings highlight the impact of tumor burden on DOX-induced cardiotoxicity mechanisms and underscore the value of tumor-bearing models in preclinical research for developing context-specific cardioprotective strategies.

Unlike conventional chronic DOX-induced cardiotoxicity models with longer treatment durations, the modeling period in this study was limited to three weeks due to ethical constraints related to tumor burden, as animals were required to be euthanized when tumor volume approached 1500 mm^3^. Despite the shortened modeling duration, cardiac dysfunction was already evident, with impaired systolic function observed in DOX-treated tumor-bearing mice compared with controls. Tissue analysis revealed structural alterations in the heart, as reflected by changes in the heart weight-to-tibia length ratio and histopathological findings based on H&E staining, indicating increased inflammatory cell infiltration. Serum levels of myocardial injury markers, including BNP and CK-MB, were also assessed. Notably, in tumor-bearing mice treated with DOX, both BNP and CK-MB levels were significantly increased, further supporting the presence of myocardial injury under tumor-bearing conditions. These findings suggest that the cardiac injury response may not only be driven by the direct toxicity of DOX but also modulated by the systemic physiological environment. Increasing evidence indicates the existence of bidirectional biochemical interactions between the heart and tumors, further complicating the relationship between cancer and cardiovascular diseases [9].

From a transcriptomic perspective, the number of DEGs induced by DOX under tumor-bearing conditions was lower than that observed in non-tumor-bearing conditions, suggesting that tumor burden may influence the cardiac transcriptional response to DOX. The reduction in DEG number may be associated with several factors. First, tumor burden is often accompanied by systemic inflammation and stress responses, which can alter the basal transcriptional state of the heart and attenuate gene expression changes in response to DOX [10,11]. In addition, tumor-associated metabolic and immune alterations may affect the overall responsiveness of the organism to pharmacological stimuli, potentially limiting the extent of transcriptional changes in cardiac tissue. Furthermore, tumor-related immune regulatory states may modulate transcriptional networks and influence the cardiac response to injury signals [12,13]. Compared with previous studies based on non-tumor-bearing models, which typically report a greater number of DEGs, the present findings suggest that DOX-induced transcriptional responses may exhibit context dependency under complex pathological conditions.

A total of 206 shared DEGs were identified between the tumor-bearing and non-tumor-bearing models. Among these, 16 genes exhibited opposite expression trends—including *Ldhc*, *Crisp2*, *Adam5*, *Prm2*, *Smcp*, *Atp8b3*, *Spz1*, *Tnp1*, *Gm32699*, *Akap3*, *Akap4*, *Pmfbp1*, *Ssu2*, *Gm17249*, *Cct8l1*, and *Clgn*—a subset predominantly involved in spermatogenesis, sperm motility, fertilization, and male germ cell development. The divergent regulation of these reproductive-related genes between the two states may reflect context-dependent modulation of germ cell-associated pathways by the tumor microenvironment. Functional enrichment analysis of shared DEGs revealed that several pathways were consistently enriched under both conditions, including NF-κB signaling, chemokine signaling, Th1/Th2 and Th17 cell differentiation, antigen processing and presentation, cell adhesion molecules, and ECM–receptor interaction. These pathways are predominantly associated with immune-inflammatory responses and intercellular communication, suggesting that DOX-driven immune dysregulation and matrix remodeling represent core mechanisms of cardiotoxicity that operate independently of the tumor-bearing state.

Further analysis of condition-specific differentially expressed genes revealed distinct mechanistic profiles. Under non-tumor-bearing conditions, DOX-associated transcriptional alterations were mainly involved in p53-mediated apoptosis, oxidative stress, and fatty acid metabolic dysregulation. This profile was consistent with the well-established mechanisms of DOX-induced DNA damage and mitochondrial dysfunction in cardiomyocytes [14,15]. Specifically, activation of the p53 pathway is closely associated with cardiomyocyte apoptosis, whereas mitochondrial dysfunction leads to the accumulation of reactive oxygen species (ROS) and disruption of energy metabolism. In contrast, under tumor-bearing conditions, condition-specific DEGs were predominantly enriched in immune-related signaling pathways, including JAK-STAT, NOD-like receptor, and Toll-like receptor pathways, suggesting that DOX may elicit a more pronounced immune response in the context of tumor-associated inflammatory priming. This difference may be associated with tumor-associated alterations in immune status, such as the systemic redistribution of immune cells and their potential infiltration into cardiac tissue. Previous studies have demonstrated that DOX can contribute to cardiac inflammation through the activation of innate immune pathways, such as cGAS–STING signaling, and the present findings further support the involvement of immune-related mechanisms under tumor-bearing conditions [16].

Taken together, these results suggest that the molecular mechanisms underlying DOX-induced cardiotoxicity may differ across physiological contexts. Specifically, non-tumor-bearing conditions appear to be more closely associated with metabolic dysregulation and direct cellular injury, whereas tumor-bearing conditions may involve a greater contribution from immune-inflammatory pathways. These differences provide a basis for further investigation into context-dependent intervention strategies, although the underlying mechanisms require further validation. Clinically, dexrazoxane remains the only agent approved for DOX cardiotoxicity. Based on our transcriptomic findings of enrichment of JAK-STAT, Toll-like receptor, and NOD-like receptor pathways in the tumor-bearing model, we propose that modulators of these signaling pathways could be explored as adjunctive therapies. However, mechanistic validation of these pathways was not performed in the present study, and therefore future work will be needed to experimentally test the therapeutic potential of targeting the tumor-associated immune-inflammatory pathways identified herein.

Several limitations of this study should be considered. First, transcriptomic analysis was performed at a single time point, which may not fully capture dynamic transcriptional changes during the progression of DOX-induced cardiotoxicity. Future studies incorporating multiple time points would help to provide a more comprehensive understanding of the temporal dynamics of these molecular events. Second, only a single tumor cell line was used in this study. Given the heterogeneity among different tumor types, particularly in metabolic and immune characteristics, further validation in additional models would strengthen and generalize the present findings.

## 4. Materials and Methods

### 4.1. Ethical Statement

This research conformed to the Guide for the Care and Use of Laboratory Animals published by the US National Institutes of Health (NIH Publication, 8th Edition, 2011). All experimental protocols were approved by the Ethics Committee of Bengbu Medical University (protocol No. 2023–171). Research staff had received specialized training in animal care and handling, including animal ethics (3R principles, local regulations) and technical skills (restraint, injection, euthanasia). The mice were fed in a specific pathogen-free environment (12 h/12 h light/darkness, 45–60% relative humidity, and 24–26 °C room temperature) with free access to food and water. The overall health status was checked by trained professionals. All efforts were made to minimize animal suffering. During the experiment, animal wellbeing was monitored every 12 h for signs of distress and endpoints. The following predefined humane endpoints were employed for euthanasia: moribund state (inability to maintain upright posture with or without labored breathing/cyanosis), or fulfillment of ≥4 criteria from: >20% weight loss, tumor volume exceeding 1500 mm^3^, anorexia, sustained hunched posture (>12 h), prostration, motility impairment, dyspnea, ruffled fur, or dehydration. Mice were euthanized by cervical dislocation after induction of anesthesia (inhalation of isoflurane (RWD, Shenzhen, China)) upon meeting humane endpoints.

### 4.2. Mouse Model Establishment

4T1 (Procell system, Wuhan, China) murine breast cancer cells were cultured in RPMI-1640 medium (Gibco (Thermo Fisher Scientific), Waltham, MA, USA) supplemented with 10% fetal bovine serum (FBS (ExCell, Suzhou, China)) and 1% penicillin/streptomycin (Biosharp-LABGIC, Beijing, China). Cells in the logarithmic growth phase were collected, adjusted to an appropriate concentration, and subcutaneously injected to establish tumor-bearing mouse models. After model establishment, mice were randomly assigned to a tumor-bearing DOX-treated group and a tumor-bearing control group (*n* = 6 per group). Both the tumor-bearing DOX-treated group and the non-tumor-bearing DOX-treated group received intraperitoneal injections of DOX (Yuanye Bio-Technology Co., Ltd, Shanghai, China) at a dose of 5 mg/kg once weekly for three consecutive weeks.

### 4.3. Echocardiographic Detection

Cardiac ultrasound examinations were performed after modeling following standardized protocols. Mice were anesthetized with 2% isoflurane via nasal inhalation and positioned supine on a temperature-controlled platform. M-mode echocardiograms were acquired using the MS-400 high-frequency transducer (30 MHz, Fujifilm VisualSonics, Inc., Toronto, ON, Canada) positioned at the parasternal short-axis view at the papillary muscle level. Cardiac architecture and functional parameters were quantified using a high-resolution small-animal ultrasound system (Visual Sonics Vevo 2100, Fujifilm VisualSonics, Inc., Toronto, ON, Canada) with the following measurements. Derived functional indices, including ejection fraction (EF) and fractional shortening (FS), were calculated using established formulas.

### 4.4. Histopathological Examination of Heart Tissue

Heart tissues were routinely collected, fixed, processed, and sectioned. Hematoxylin and eosin (H&E) staining was performed on 4 μm paraffin sections. After deparaffinization and rehydration, sections were stained with Mayer’s hematoxylin, differentiated in 0.5% acid alcohol, counterstained with eosin Y, and mounted with resin. Each group consisted of 6 samples. Three regions were randomly selected from each sample for histopathological observation. Heart tissues were harvested and fixed in 4% paraformaldehyde, followed by paraffin embedding. Tissue sections were prepared and deparaffinized with xylene and rehydrated through a graded ethanol series. The sections were then incubated with fluorescently labeled wheat germ agglutinin (WGA) (Servicebio, Wuhan, China) according to the manufacturer’s instructions to visualize the cell membrane. Nuclei were counterstained with DAPI. Images were captured using a fluorescence microscope. Cardiomyocyte cross-sectional area (CSA) was measured using ImageJ (version 1.54f, National Institutes of Health, Bethesda, MD, USA). For each sample, multiple fields were randomly selected, and at least 100 cardiomyocytes were analyzed.

### 4.5. Detection of Serum Cardiac Injury Markers by ELISA

Serum N-terminal pro-brain natriuretic peptide (NT-proBNP) levels were analyzed using a commercial enzyme-linked immunosorbent assay (ELISA) kit (Cat# JL11641, Jianglai Biotechnology, Shanghai, China) according to the manufacturer’s instructions. Creatine kinase-MB (CK-MB) levels in mice were determined using a commercial ELISA kit (Cat# D721065, Sangon Biotech, Shanghai, China) according to the manufacturer’s instructions.

### 4.6. Transcriptome Sequencing

Total RNA was extracted from target tissues and subjected to quality assessment. mRNA was enriched using oligo (dT) magnetic beads, fragmented, and reverse-transcribed into double-stranded cDNA. After end repair and adapter ligation, the libraries were amplified by PCR and purified using magnetic beads. High-throughput sequencing was performed on an Illumina sequencing platform. Raw sequencing reads in FASTQ format were processed using Cutadapt (v1.9.1) to remove technical sequences and low-quality bases. The mRNA expression profiles were deposited in the Gene Expression Omnibus (GEO) database (GSE324519). The threshold for significant enrichment was set at *p* < 0.05. Gene Ontology (GO) enrichment analysis and Kyoto Encyclopedia of Genes and Genomes (KEGG) pathway enrichment analysis of DEGs were performed.

### 4.7. Validation of mRNA Expression Levels by qPCR

Total RNA was extracted from heart tissues using TRIzol reagent (Cat# G3013, Servicebio, Servicebio Technology Co., Ltd., Wuhan, Hubei, China). Complementary DNA (cDNA) was synthesized using the PrimeScript™ RT Reagent Kit (Cat# R333-01,Vazyme Biotech Co., Ltd., Nanjing, Jiangsu, China). Quantitative real-time PCR (qRT-PCR) was performed using SYBR Green PCR Master Mix (Cat# 11201ES03, Yeasen Biotechnology (Shanghai) Co., Ltd., Shanghai, China). The primer sequences were as follows: *Mfap4*, 5′-AGTGCTGAGGAGGATGGCTA-3′ and 5′-CTTCTGGCCACTGTGGTAGG-3′; *ITGB6*, 5′-CTCATTCCTGGAGCAACCGT-3′ and 5′-CACCTCAGACCGCAGTTCTT-3′; *UCP3*, 5′-CATCGCCAGGGAGGAAGGAG-3′ and 5′-GGTCACCATCTCAGCACAGTTG-3′; *Gapdh*, 5′-AGGTCGGTGTGAACGGATTTG-3′ and 5′-TGTAGACCATGTAGTTGAGGTCA-3′ (Sangon Biotech, Shanghai, China). Relative mRNA expression levels were normalized to GAPDH and calculated using the 2^−ΔΔCt^ method.

### 4.8. Statistical Analysis

Statistical analysis was performed using GraphPad Prism 8.0 (GraphPad Software, Boston, MA, USA). Data are presented as mean ± SD. For comparisons among multiple groups, two-way analysis of variance (ANOVA) was applied, while comparisons between two groups were performed using Student’s t-test. A significance threshold was set at *p* < 0.05.

## 5. Conclusions

In this study, we systematically compared the phenotypic characteristics and transcriptomic differences in DOX-induced cardiotoxicity in tumor-bearing and non-tumor-bearing mouse models. The findings indicate that although DOX induces cardiac structural and functional impairment in both models, the phenotypic manifestations and molecular response patterns differ under tumor-bearing conditions. At the histological and serological levels, tumor burden appears to modulate the phenotypic features of DOX-induced cardiac injury. At the transcriptomic level, shared differentially expressed genes suggest the presence of common underlying injury responses, whereas condition-specific DEGs and their functional enrichment further indicate that tumor burden may reshape the cardiac transcriptional response, particularly with respect to the involvement of immune-related signaling pathways and cellular response processes. Taken together, these findings suggest that tumor burden represents a critical factor influencing DOX-induced cardiotoxicity models and should be carefully considered in mechanistic studies and model selection. This study provides a methodological reference for the rational design of animal models and transcriptomic investigations of DOX-induced cardiotoxicity.

## Data Availability

The RNA-seq data have been deposited in the Gene Expression Omnibus (GEO) database under accession number GSE324519. Other data supporting the findings of this study are available from the corresponding author upon reasonable request.
